# Dynamical law of the phase interface motion in the presence of crystals nucleation

**DOI:** 10.1038/s41598-022-15137-2

**Published:** 2022-06-29

**Authors:** Liubov V. Toropova, Dmitri V. Alexandrov

**Affiliations:** 1 Otto-Schott-Institut für Materialforschung, Friedrich-Schiller-Universität-Jena, 07743 Jena, Germany; 2 Laboratory of Mathematical Modeling of Physical and Chemical Processes in Multiphase Media, Department of Theoretical and Mathematical Physics, Ural Federal University, Lenin Ave., 51, Ekaterinburg, 620000 Russian Federation; 3 Laboratory of Multi-Scale Mathematical Modeling, Department of Theoretical and Mathematical Physics, Ural Federal University, Lenin Ave., 51, Ekaterinburg, 620000 Russian Federation

**Keywords:** Applied mathematics, Theoretical particle physics

## Abstract

In this paper, we develop a theory of solid/liquid phase interface motion into an undercooled melt in the presence of nucleation and growth of crystals. A set of integrodifferential kinetic, heat and mass transfer equations is analytically solved in the two-phase and liquid layers divided by the moving phase transition interface. To do this, we have used the saddle-point method to evaluate a Laplace-type integral and the small parameter method to find the law of phase interface motion. The main result is that the phase interface *Z* propagates into an undercooled melt with time *t* as $Z(t)=\sigma \sqrt{t}+\varepsilon \chi t^{7/2}$ with allowance for crystal nucleation. The effect of nucleation is in the second contribution, which is proportional to $t^{7/2}$ whereas the first term $\sim \sqrt{t}$ represents the well-known self-similar solution. The nucleation and crystal growth processes are responsible for the emission of latent crystallization heat, which reduces the melt undercooling and constricts the two-phase layer thickness (parameter $\chi <0$).

## Introduction

The dynamics of crystallization fronts propagation have attracted the attention of researchers for more than 130 years, starting with Stefan’s famous works on the freezing of water with a flat front^1,2^. Nowadays, problems with a moving boundary separating different phases of the aggregate state of matter bear his name. The rich variety of nonlinear dynamics of interfacial boundaries has attracted essential interest in applied mathematics, which deals with various moving boundary problems in geophysics, materials science, nonlinear physics, and heat and mass transfer theory (see, among others,^3–6^).

It is well-known that the phase transition boundary in Stefan’s problem moves according to the self-similar law (it is proportional to the square root of time). Such a law is preserved even in the presence of an extended phase transition region separating purely solid and liquid phases in binary^7^ and three-component^8,9^ melts. For example, in the case of binary melts, there is one two-phase region and two interphase boundaries moving in proportion to the square root of time *t*^7^. In the case of ternary melts, there are two two-phase regions (main and cotectic) and three interphase boundaries, moving according to the law $\sim \sqrt{t}$ with various parabolic growth rate constants^9^. Note that self-similar solutions take place only in the semi-infinite region whose boundary temperature is kept constant. At a given heat flow at this boundary or its active cooling by air or water flow, there is no self-similarity of the phase interface motion^10^. There is also no self-similarity of motion of the interfacial boundary (boundaries) when considering nonlinear heat conduction and impurity diffusion equations, heat and mass sources in these equations, heat exchange with the environment, melt convection, kinetics, stochastic fluctuations as well as other factors (see, among others^11–16^). In this study, we derive the law for phase interface motion with allowance for nucleation and growth of crystals in front of it. These effects significantly change the previously known self-similar law due to the thinning of the two-phase layer as a result of additional latent heat released by the growing crystals. The theory developed can be used in describing the propogation of various solid/liquid interfaces in metastable media and experimental data on Al-rich Al–Ni alloys showing an anomalous solidification behaviour: the solid–liquid interface velocity slows down as the undercooling increases^17,18^.

## The model

**Figure 1:**
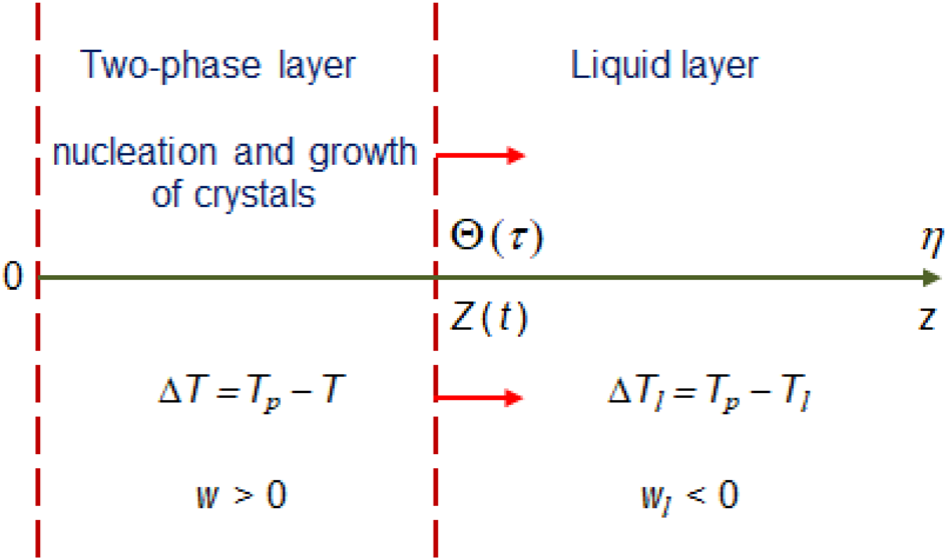
A schematic illustration of crystallization with an undercooled layer where nucleation and growth of crystallites occur.

First of all, let’s assume a one-component melt filling a semi-space $\eta >0$ (Fig. 1). We will also assume that the initial temperature $T_l$ of the melt exceeds its crystallization temperature $T_p$ at the time $\tau =0$. The temperature $T =T_0$ at the solid wall $\eta =0$ then jumps to a value of $T < T_p$. This generates the melt undercooling $\Delta T = T_p - T$ propagating into the melt phase $0<\eta <\Theta (\tau )$. Here, $\Theta (\tau )$ stands for the interface boundary with temperature $T = T_l = T_p$ (*T* and $T_l$ denote the temperatures in the moving domains $0<\eta <\Theta (\tau )$ and $\eta > \Theta (\tau )$). The undercooled domain $0<\eta <\Theta (\tau )$ is filled with nucleating particles whose nucleation rate is $I(\Delta T)$ ($\Delta T =0$ at the interface $\eta =\Theta (\tau )$). As a result of crystal growth, the melt undercooling is partially reduced due to the release of latent heat of crystallisation. This phenomenon is the reason for slowing down the interfacial boundary movement into the liquid melt.

For the sake of simplicity, we consider the growth of an ensemble of spherical particles whose distribution function $\phi (\eta ,r,\tau )$ satisfies the first-order kinetic equation. In addition, we have the thermal conductivity equations in both the domains with allowance for the heat source in the two-phase region as a result of crystal growth. The governing equations read as

1 $$\begin{aligned}\frac{\partial \phi }{\partial \tau }+\frac{\partial }{\partial r}\left( \frac{dr}{d\tau }\phi \right) =0,\ 0<\eta <\Theta (\tau ),\ r>0,\ \tau >0, \end{aligned}$$

2 $$\begin{aligned}\rho c \frac{\partial T}{\partial \tau } = \lambda \frac{\partial ^2T}{\partial \eta ^2}+\frac{4\pi Q_v}{3}\frac{\partial }{\partial \tau }\int \limits _0^\infty r^3\phi dr ,\ 0<\eta <\Theta (\tau ),\ \tau >0, \end{aligned}$$

3 $$\begin{aligned}\rho _l c_l \frac{\partial T_l}{\partial \tau } = \lambda _l \frac{\partial ^2T_l}{\partial \eta ^2} ,\ \eta>\Theta (\tau ),\ \tau >0. \end{aligned}$$

Here $Q_v$ is the latent heat of phase transition, $\rho$ and $\rho _l$ are the densities of two-phase and melt phases, *c* and $c_l$ are their heat capacities, and $\lambda$ and $\lambda _l$ are their heat conductivity coefficients, respectively (subscript *l* denotes liquid region).

These equations should be supplemented with the conditions at $r=0$, $\tau =0$, $\eta =0$, $\eta \rightarrow \infty$, and the phase-transition boundary $\eta =\Theta (\tau )$, of the form of

4 $$\begin{aligned} \frac{dr}{d\tau }\phi= & {} I(\Delta T) ,\ r=0,\ \tau >0;\ \phi =0,\ \tau =0,\ 0<\eta <\Theta (\tau ); \end{aligned}$$

5 $$\begin{aligned} T= & {} T_0,\ \eta =0,\ \tau>0 ;\ T_l \rightarrow T_\infty , \ \eta \rightarrow \infty , \tau >0; \end{aligned}$$

6 $$\begin{aligned} T= & {} T_l = T_p,\ \frac{\partial T}{\partial \eta }= \frac{\partial T_l}{\partial \eta },\ \eta = \Theta (\tau ),\ \tau >0 ; \ T_l = T_\infty , \ \tau =0. \end{aligned}$$

Let us especially underline that the first expression (4) gives the flux of nucleating crystals that appear within the two-phase region.

We consider the growth rate of single crystals as a power function of melt undercooling^19–22^.

7 $$\begin{aligned} \frac{dr}{d\tau }= \beta _k \left( \Delta T\right) ^n, \end{aligned}$$

where $\beta _k$ and *n* are constants. These parameters can be found from experimental data on crystal growth in a certain undercooled liquid.

For the sake of simplicity, we consider the case when the nucleation rate depends only on the melt undercooling. Dealing the case of the Weber–Volmer–Frenkel–Zel’dovich (WVFZ) nucleation kinetics, we have^23^

8 $$\begin{aligned} I(\Delta T) = I_*\exp \left[ -p\left( \frac{\Delta T_0}{\Delta T}\right) ^2 \right] , \end{aligned}$$

where $\Delta T_0$ represents the initial melt undercooling. Another frequently used kinetics is Meirs power law^24–26^

9 $$\begin{aligned} I(\Delta T) = I_*\left( \Delta T\right) ^p. \end{aligned}$$

It is significant that the constant parameters $I_*$ and *p* entering expressions (8) and (9) are different.

## Analytical solutions to moving-boundary problem

The aforementioned model represents a set of integrodifferential equations, initial and boundary conditions with a moving phase interface $\Theta (\tau )$. Below we develop a method to its analytical solution, which is based on the saddle-point technique to evaluate a Laplace-type integral^27,28^ and the small parameter technique to solve a nonlinear heat transfer equation for the melt undercooling.

To simplify the matter, we use dimensionless parameters as follows

10 $$\begin{aligned} \begin{array}{l} \displaystyle w=\frac{\Delta T}{\Delta T_0},\ w_l=\frac{\Delta T_l}{\Delta T_0},\ F=\ell ^4\phi ,\ s=\frac{r}{\ell },\ z=\frac{\eta }{\ell },\\ \displaystyle Z=\frac{\Theta }{\ell } ,\ t=\frac{\tau }{\tau _o},\ b=\frac{4\pi Q_v}{3\rho c \Delta T_0},\ \gamma = \frac{\lambda \tau _o}{\rho c \ell ^2},\ I_0 = I\left( \Delta T_0 \right) , \\ \displaystyle \tau _o = \frac{\ell }{\beta _k\left( \Delta T_0\right) ^n},\ \ell =\left( \frac{\beta _k\left( \Delta T_0\right) ^n}{I_0} \right) ^{1/4}, \end{array} \end{aligned}$$

where $\Delta T_0 =T_p-T_0$ and $\Delta T_l =T_p-T_l$ represent the initial and current melt undercoolings.

The dimensionless distribution function *F* of growing crystals in a two-phase region is defined by the following problem (this problem can be easily obtained from Eqs. (1), (4), (7)–(9) rewritten in dimensionless form)

11 $$\begin{aligned}\frac{\partial F}{\partial t}+w^n\frac{\partial F}{\partial s} =0,\ s>0,\ t>0, \end{aligned}$$

12 $$\begin{aligned}F=\frac{1}{w^n}\exp \left[ pg(w) \right] ,\ s=0;\ F=0,\ t=0, \end{aligned}$$

where

$$\begin{aligned} g(w)=g\left( z,t \right) = \left\{ \begin{array}{l} 1-w^{-2} ,\ \mathrm{WVFZ\ nucleation\ mechanism} \\ \ln w ,\ \mathrm{Meirs\ nucleation\ mechanism}. \end{array} \right. \end{aligned}$$

Applying the Laplace integral transform to the problem (11) and (12) with respect to *t*, we find the particle-radius distribution function of the form

13 $$\begin{aligned} F(z,s,t)= \varphi \left( x(z,t)-s \right) \mathrm{Heav}\left( x(z,t)-s \right) , \end{aligned}$$

where

14 $$\begin{aligned} \varphi (z,t)=\frac{1}{w^n}\exp \left( pg(z,t) \right) ,\ x(z,t)=\int \limits _0^t w^n\left( z,t_1 \right) dt_1, \end{aligned}$$

and $\mathrm{Heav} (\cdot )$ is the Heaviside function.

Now substituting dimensionless variables and parameters (10) into Eqs. (2), (3) and boundary conditions (5) and (6), we obtain

15 $$\begin{aligned}\frac{\partial w}{\partial t}=\gamma \frac{\partial ^2w}{\partial z^2}-b \frac{\partial }{\partial t} \int \limits _0^t h(z,\nu ,t)\exp \left( pg(z,\nu ) \right) d\nu ,\ 0<z<Z(t),\ t>0, \end{aligned}$$

16 $$\begin{aligned}\frac{\partial w_l}{\partial t}=\gamma \frac{\partial ^2w_l}{\partial z^2} ,\ z>Z(t),\ t>0, \end{aligned}$$

17 $$\begin{aligned}w = 1,\ z =0,\ t>0 ;\ w_l \rightarrow w_\infty = \frac{T_p-T_\infty }{\Delta T_0} , \ z \rightarrow \infty ,\ t >0; \end{aligned}$$

18 $$\begin{aligned}w = w_l = 0,\ \frac{\partial w}{\partial z}= \frac{\partial w_l}{\partial z},\ z = Z (t),\ t >0 ; \ w_l = w_\infty , \ t =0, \end{aligned}$$

where $w>0$ in the two-phase layer, and $w_l<0$ in the liquid layer. Also, for the sake of simplicity, we assume that $\lambda = \lambda _l$, $\rho = \rho _l$, and $c=c_l$. Let us especially emphasize that the new variable $\nu$ has been used in Eq. (15) so that $x(z,\nu )=x(z,t)-s$, and

$$\begin{aligned} h(z,\nu ,t)= \left[ x(z,t)- x(z,\nu ) \right] ^3. \end{aligned}$$

To evaluate the integral in the r.h.s. of Eq. (15) we apply the saddle-point technique for a Laplace-type integral^27,28^. The $\nu$-derivatives of the function *g* are negative for both nucleation kinetics under consideration since the melt undercooling *w* is a decreasing function of time: $\partial g/\partial \nu = (dg/dw)\partial w/\partial \nu <0$. It means that the maximum point of the function *g* lies at the left boundary at $\nu =0$. To calculate the first non-zero derivative of $g(z,\nu )$ with respect to $\nu$ we use the same Eq. (15). It is not difficult to show that the first three derivatives are zero and only the fourth one is non-zero for both nucleation kinetics. Namely, $g^{(4)}=-12b$ (WVFZ) and $g^{(4)}=-6b$ (Meirs) at $\nu =0$. Now keeping in mind only the main contribution in the asymptotic expansion of the integral (15), we come to Ref.^27,28^

19 $$\begin{aligned} \frac{\partial u}{\partial t}= & {} \gamma \frac{\partial ^2u}{\partial z^2}- \varepsilon y^2u^n ,\ 0<z<Z(t),\ t>0, \end{aligned}$$

20 $$\begin{aligned} \frac{\partial u_l}{\partial t}= & {} \gamma \frac{\partial ^2u_l}{\partial z^2},\ z>Z(t),\ t>0, \end{aligned}$$

where

21 $$\begin{aligned} u=Aw,\ u_l=Aw_l,\ A=\frac{3b^{3/4}\Gamma (1/4)}{4 p^{1/4}}\left( \frac{4}{\mu } \right) ^{1/4},\ y(z,t)=\int \limits _0^t u(z,t_1)dt_1, \end{aligned}$$

$\varepsilon =A^{-n}$; $\mu =2$ and $\mu =1$ for the WVFZ and Meirs mechanisms.

Our estimations show that $\varepsilon \ll 1$ for typical metallic melts^29,30^. This fact enables us seeking for the solution of Eq. (19) expanding the rescaled undercooling *u* in a series in small parameter $\varepsilon$ as

22 $$\begin{aligned} u=u_0+\varepsilon u_1+ \ldots ,\ Z=Z_0+\varepsilon Z_1+\ldots \end{aligned}$$

Substituting (22) into (19) and (20), expanding conditions at $z=Z(t)$ in series and equating terms with the same power of $\varepsilon$, we arrive at the following form of solutions

23 $$\begin{aligned} u_0=\Phi _0(\zeta ),\ u_1 = \Phi _1(\zeta )t^3,\ \zeta = \frac{z}{\sqrt{t}},\ Z_0(t)=\sigma \sqrt{t},\ Z_1(t)=\chi t^{7/2}, \end{aligned}$$

where $\sigma$ and $\chi$ represent the constant parameters characterizing the interface position *Z*(*t*). The functions $\Phi _0(\zeta )$ and $\Phi _1(\zeta )$ satisfy the following equations and boundary conditions

24 $$\begin{aligned}\gamma \frac{d^2\Phi _0}{d\zeta ^2}=-\frac{\zeta }{2}\frac{d\Phi _0}{d\zeta },\ \gamma \frac{d^2\Phi _1}{d\zeta ^2}=\Psi (\zeta )-\frac{\zeta }{2}\frac{d\Phi _1}{d\zeta },\ \gamma \frac{d^2u_l}{d\zeta ^2}=-\frac{\zeta }{2}\frac{du_l}{d\zeta }, \end{aligned}$$

25 $$\begin{aligned}\Phi _0=A,\ \Phi _1=0,\ \zeta =0;\ u_l \rightarrow Aw_\infty ,\ \zeta \rightarrow \infty ; \end{aligned}$$

26 $$\begin{aligned}\Phi _0=0,\ \Phi _1+\chi \frac{d\Phi _0}{d\zeta }=0,\ u_l=0,\ \frac{d\Phi _0}{d\zeta }=\frac{du_l}{d\zeta },\ \frac{d\Phi _1}{d\zeta }=0,\ \zeta = \sigma , \end{aligned}$$

where

$$\begin{aligned} \Psi (\zeta ) = 4K^2(\zeta )\zeta ^4 \Phi _0^n(\zeta )\ \mathrm{and}\ K(\zeta ) = \int \limits _\zeta ^\infty \frac{\Phi _0(\zeta _1)}{\zeta _1^3}d\zeta _1. \end{aligned}$$

The analytical solution to the model (24)–(26) takes the form

27 $$\begin{aligned} \Phi _0(\zeta )= & {} A\left[ 1-\frac{\displaystyle \mathrm{erf} \left( \frac{\zeta }{2\sqrt{\gamma }} \right) }{\displaystyle \mathrm{erf} \left( \frac{\sigma }{2\sqrt{\gamma }} \right) }\right] ,\ \Phi _1(\zeta ) = \int \limits _0^\zeta \left( \Omega (\zeta _1) -\Omega (\sigma )\right) \exp \left( -\frac{\zeta _1^2}{4\gamma } \right) d\zeta _1 , \end{aligned}$$

28 $$\begin{aligned} u_l(\zeta )= & {} Aw_\infty \left[ 1-\frac{\displaystyle \mathrm{erfc} \left( \frac{\zeta }{2\sqrt{\gamma }} \right) }{\displaystyle \mathrm{erfc} \left( \frac{\sigma }{2\sqrt{\gamma }} \right) }\right] ,\ \Omega (\zeta ) = \gamma ^{-1}\int \limits _0^\zeta \Psi (\zeta _1)\exp \left( \frac{\zeta _1^2}{4\gamma } \right) d\zeta _1. \end{aligned}$$

Here, parameters $\sigma$ and $\chi$ satisfy the equations

29 $$\begin{aligned}\mathrm{erfc} \left( \frac{\sigma }{\sqrt{4\gamma }} \right) +w_\infty \mathrm{erf} \left( \frac{\sigma }{\sqrt{4\gamma }} \right) =0,\ \end{aligned}$$

30 $$\begin{aligned}\chi = A^{-1}\sqrt{\pi \gamma }\mathrm{erf} \left( \frac{\sigma }{\sqrt{4\gamma }} \right) \exp \left( \frac{\sigma ^2}{4\gamma } \right) \Phi _1(\sigma ). \end{aligned}$$

Let us especially note that expressions (27) and (28) define the melt undercooling $w>0$ within the two-phase region at $0<z<Z(t)$ and the undercooling $w_l<0$ in pure melt at $z>Z(t)$.

## Behaviour of solutions

**Figure 2:**
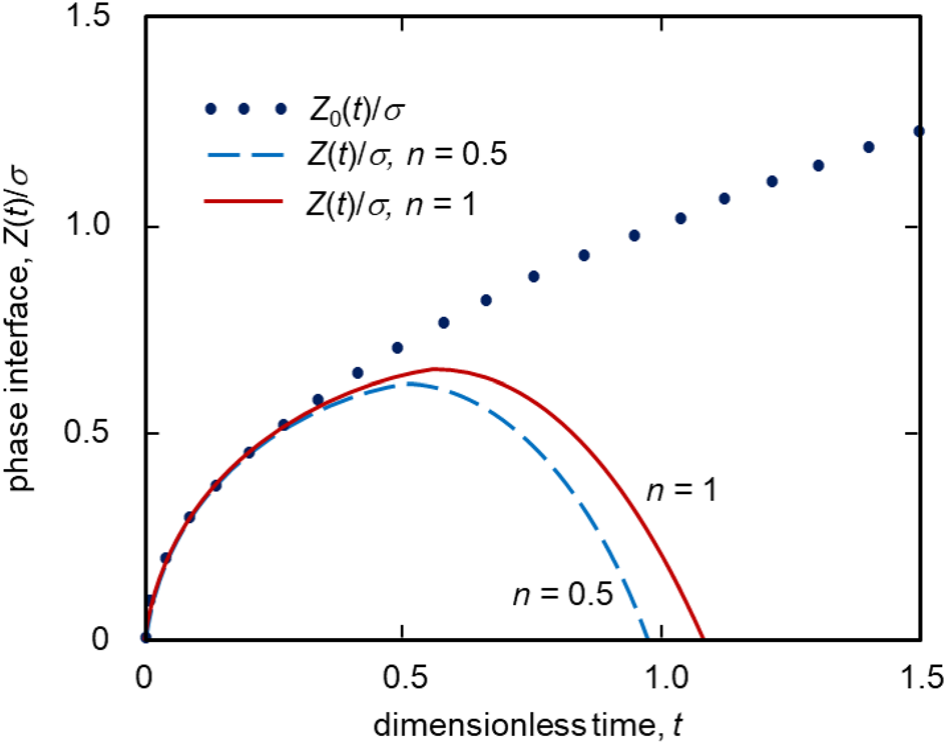
Rescaled phase interface $Z(t)/\sigma$ as a function of dimensionless time *t* at different *n*. The dotted and solid curves respectively show zero and first approximations of the interface position. Physical parameters used for calculations are^30^: $\rho = 7\times 10^3$ kg m$^{-3}$, $c=426.77$ J kg$^{-1}$ K$^{-1}$, $Q_v=1.58 \times 10^{10}$ J m$^{-3}$, $\Delta T_0 =400$ K, $p=10$, $w_\infty =-1$, $\mu =2$, $\lambda =41.84$ J s$^{-1}$ m$^{-1}$ K$^{-1}$, $\beta _k=10^{-4}$ m s$^{-1}$ K$^{-n}$, $\ell =4.47\times 10^{-10}$ m, $\sigma =8.44 \times 10^4$, $\tau _o=1.12 \times 10^{-4}$ s. The phase interface as a function of time *t* is plotted for $n=0.5$ (dashed line) and $n=1$ (solid line). The dotted line representing the main contribution $Z_0(t)/\sigma$ is independent of *n*.

**Figure 3:**
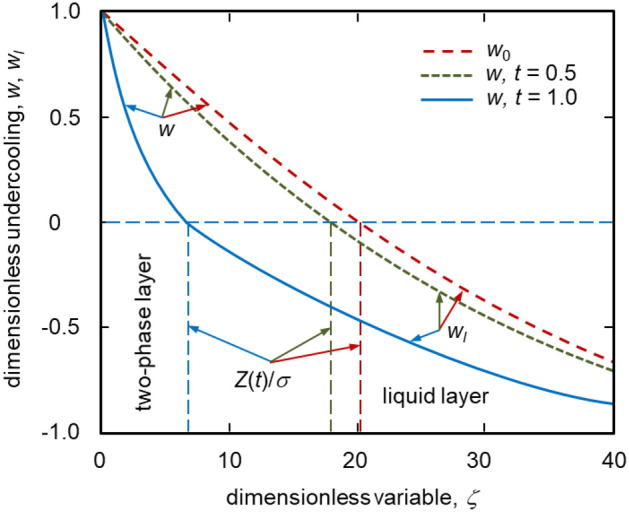
Dimensionless undercooling in the two-phase ($w>0$) and liquid ($w_l<0$) layers as a function of dimensionless coordinate $\zeta$. The dashed line shows zero approximation $w_0$ whereas the dotted and solid curves illustrate the first approximation *w* at different times *t*. The vertical lines illustrate the interface positions $Z(t)/\sigma$ dividing two-phase and liquid layers. The dimensionless undercooling as a function of self-similar varaible $\zeta$ is shown for $t=0.5$ (dotted line) and $t=1$ (solid line). The dashed line representing the main contribution $w_0$ is independent of *t*. All curves are plotted for $n=1$.

**Figure 4:**
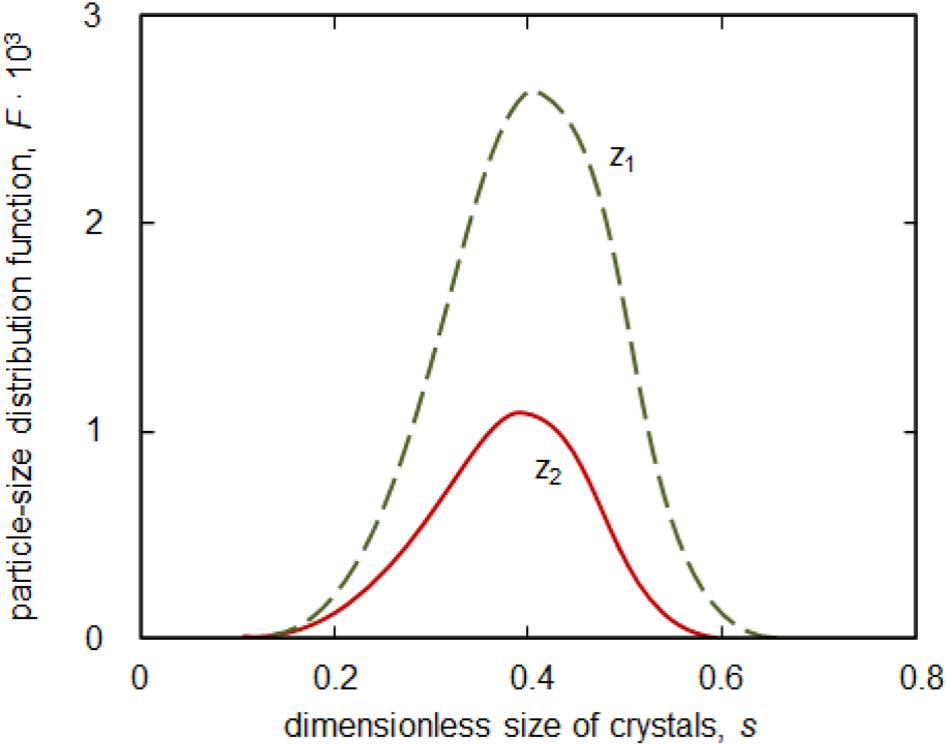
Dimensionless particle-size distribution function *F*(*s*) at different dimensionless coordinates *z* in the two-phase layer $z_1=0.9\times 10^{4}$ ($w=0.5$) and $z_2=10^{4}$ ($w=0.646$) at $t=1$. The interface position $Z=1.694 \times 10^{4}$.

Our analytical solutions are illustrated in Figs. 2, 3 and 4 for parameters typical for metallic melts^30^. First of all, phase interface dynamics, shown in Fig. 2 with allowance for nucleation and growth of crystals, essentially differ from the case without particles in a two-phase layer. This purely frontal case demonstrated by the dotted curve in Fig. 2 is described by the law of the square root of time, i.e. $Z_0(t)\sim \sqrt{t}$.

Such dynamics are the property of so-called self-similar crystallization processes (see, among others^7,31^). Nucleation and growth of solid particles within the two-phase layer change this dynamical law drastically. From a certain point in time, the law of motion of the phase interface becomes a decreasing function of time. This is caused by the fact that growing crystals produce the latent heat of phase transition, which partially reduces the undercooling in a two-phase layer and, thus, its thickness. In addition, the power of nucleation rate *n* significantly affects the movement of the phase transition interface *Z*(*t*) (compare the solid and dashed curves in Fig. 2). The greater *n* (higher crystal growth rate according to expression (7)) the greater the two-phase layer thickness. Moreover, increase of *n* shifts the maximum point of phase transition boundary *Z*(*t*) towards higher values of time *t*, i.e. the compression of the two-phase layer occurs later with increasing *n*.

Figure 3 illustrates the melt undercooling in the two-phase ($w>0$) and liquid ($w_l<0$) layers. As is easily seen, nucleation and crystal growth processes substantially constrict a two-phase layer and accelerate its desupercooling dynamics when time *t* increases (compare the green dotted and blue solid curves in Fig. 3 shown for different time instants *t*). In addition, the melt undercooling in liquid becomes lower with increasing $\zeta$ and *t*. As this takes place, the first correction $w_1=u_1/A$ to the main contribution $w_0=u_0/A$ substantially influences the desupercooling dynamics as compared with $w_0$. Such a dynamics could be the key to explain the anomalous U-shape behaviour of the “recalescence front velocity— melt undercooling” curve in Al-rich Al–Ni alloys^17,18^.

The particle-radius distribution function (13) at different points *z* in the two-phase layer is shown in Fig. 4 at a certain point in time. As is easily seen, this function represents a bell-shaped curve decreasing its amplitude with increasing the spatial coordinate *z* in a two-phase region (when approaching the two-phase layer—liquid phase boundary). This is due to the fact that the melt undercooling increases with decreasing *z* (when approaching the two-phase layer boundary $z=0$). Such a bell-shaped behaviour is in agreement with typical particle-radius distribution in undercooled and supersaturated liquids (see, among others, recently published review on nucleation^32^).

## Conclusion

In summary, we develop a theory of solid/liquid phase interface motion in the presence of nucleation and particle growth processes in an undercooled layer. This layer together with evolving crystals, which partially compensate for the undercooling, propagates into pure melt with the velocity depending on crystal growth and nucleation rates. The main result of our study is the dynamical law of the phase interface boundary motion $Z(t)=\sigma \sqrt{t}+\varepsilon \chi t^{7/2}$ (two coefficients $\sigma$ and $\chi$ are found analytically). This law substantially differs from the case without nucleation and growth of crystals, which defines the phase interface as $Z_0(t)=\sigma \sqrt{t}$. The negative coefficient $\chi$ leads to the narrowing of an undercooled layer starting from a certain point in time. This is caused by the effect of latent heat emission, which reduces the melt undercooling and constricts its spatial size (the two-phase layer thickness).

The present theory can be extended to take into account fluctuations in the growth rates of individual particles in an undercooled two-phase layer. This effect raises the order of the kinetic equation in the spatial variable (see, among others^32,33^). Such consideration can modify the law of motion of the phase interface. However, the slowing down of its motion detected in this paper in comparison with the self-similar case ($Z_0(t)=\sigma \sqrt{t}$) should be preserved, since it is caused by the compensation of undercooling due to the release of the latent crystallization heat.

## Data Availability

All data generated or analysed during this study are included in this published article.

## Latin symbols

*c*: Heat capacity of two-phase zone, J kg$^{-1}$ K$^{-1}$
$c_l$: Heat capacity of melt phase, J kg$^{-1}$ K$^{-1}$
*F*: Dimensionless distribution function, –
*I*: Nucleation rate, m s$$^{-1}$$
$I_*$: Nucleation rate factor, m$^{-3}$ s$^{-1}$ (WVFZ) or m$^{-3}$ s$^{-1}$ K$^{-p}$ (Meirs)
$$\ell$$: Characteristic lenght, m
*n*: Power of nucleation rate, –
*p*: Nucleation rate parameter, –
$Q_v$: Latent heat of phase transition, J m$^{-3}$
*r*: Crystal radius, m
*t*: Dimensionless time, –
$$T_l$$: Melt temperature, K
$$T_p$$: Crystallization temperature, K
$T_{\infty }$: Temperature far from the solid/liquid interface, K
$$T_0$$: Initial temperature, K
$$\Delta T$$: Melt undercooling, K
*w*: Dimensionless undercooling in two-phase layer, –
$w_l$: Dimensionless undercooling in liquid, –
$w_{\infty }$: Dimensionless undercooling far from the solid/liquid interface, –
*z*: Dimensionless spatial coordinate , –
*Z*: Dimensionless interface coordinate, –

## Greek symbols

$$\beta _k$$: Kinetic parameter, m s$$^{-1}$$ K$$^{-n}$$
$\Gamma$: Euler gamma function, –
$\zeta$: Self-similar varaible, –
$\eta$: Dimensional spatial coordinate , m
$\Theta$: Dimensional interface coordinate, m
$\lambda$: Heat conductivity coefficient in two-phase layer, J s$^{-1}$ m$^{-1}$ K$^{-1}$
$\lambda _l$: Heat conductivity coefficient in liquid, J s$^{-1}$ m$^{-1}$ K$^{-1}$
$\rho$: Density of two-phase layer, kg m$^{-3}$
$\rho _l$: Density of liquid phase, kg m$^{-3}$
$\sigma$: Parabolic growth rate constant, –
$$\tau$$: Dimensional time, s
$$\tau _o$$: Characteristic time, s
$\phi$: Dimensional distribution function, m$^{-4}$
$\chi$: Growth rate constant, –

## Abbreviations

Heav: Heaviside function
WVFZ: Weber–Volmer–Frenkel–Zel’dovich nucleation kinetics
